# Supplementary material for: ZnO/Graphene Composite from Solvent-Exfoliated Few-Layer Graphene Nanosheets for Photocatalytic Dye Degradation under Sunlight Irradiation
Source: Micromachines (Basel). 2023 Jan 12;14(1):189. doi: 10.3390/mi14010189 (PMC9860591; doi:10.3390/mi14010189)
Supplement: Supplementary file 1 [file micromachines-14-00189-s001.zip › micromachines-2025261-supplementary.pdf]

## Supplementary Information

## ZnO/graphene composite from solvent-exfoliated few-layer graphene nanosheets for photocatalytic dye degradation under sunlight irradiation

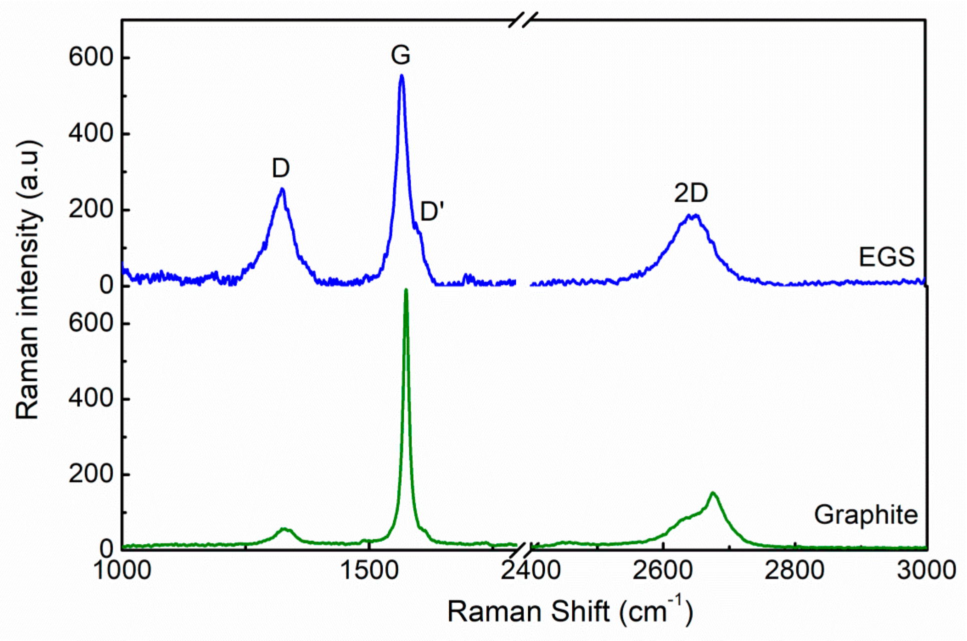


Fig.S1. Micro-Raman spectra of exfoliated graphene sheets (EGS) and graphite powder

Micro-Raman spectra of the exfoliated graphene sheet and graphite powder are shown in Fig.S1. The characteristic Raman bands of graphite powder were obtained at ~1329 (D band), ~1575 (G band) and 2675 $cm^{-1}$ (2D band). Raman bands of exfoliated graphene are shifted to low wavenumber side with respect to that of graphite powder. The D, G and 2D bands are occurred at ~1323, ~1567 and ~2645 $cm^{-1}$, respectively. The broad asymmetric 2D band of graphite changed into a sharper, high intense low frequency peak, when the interaction between the AB stacking has been broken during the exfoliation. Increase in the intensity of D and D’ band after exfoliation may be attributed to the new edges created by the exfoliation of graphite.

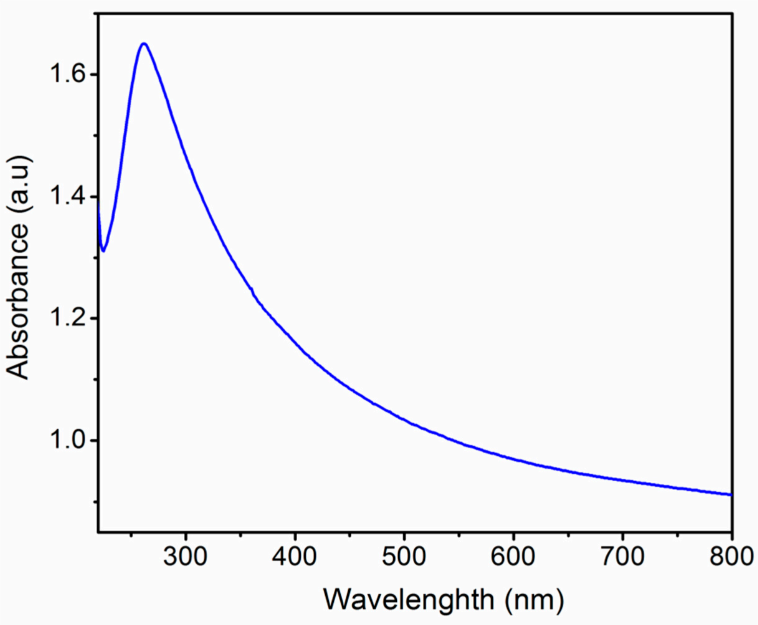


Fig.S2. UV-Vis absorption spectrum of graphene dispersion

The absorption peak around 270 nm, due to the π-π* transition of graphitic sheets which supports the exfoliation of graphene in 1,2-Dichloroethane. The concentration of the graphene dispersed in the solvents after centrifugation was estimated by filtering the dispersion using the Nylon membrane filter (pore size 0.2 μm). The filtered graphene was heated in vacuum oven at 70 °C for 22 h. The concentration of graphene dispersed in the solvent was estimated from the weight difference of the membrane filter before and after the filtration. The calculated graphene concentration is 124 μg/ml which comparatively higher than that achieved after 8 h sonication of graphite in O-DCB and also the concentration of graphene exfoliated in DMF with and without addition of naphthalene.

A few layer graphene sheets were evidenced from the TEM and AFM micrograph as shown in below figure FS3.

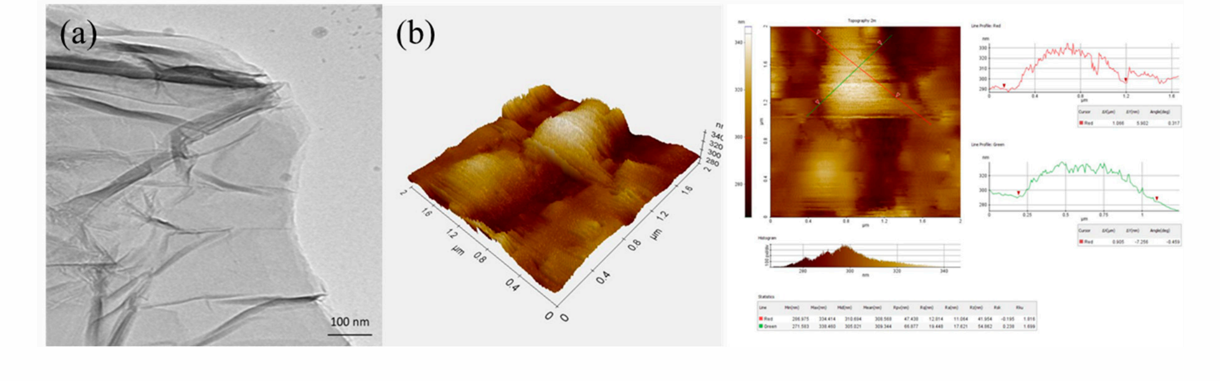

Fig.S3. TEM micrograph of graphene nanosheets.
